# Bispecific Antibodies as a Development Platform for New Concepts and Treatment Strategies

**DOI:** 10.3390/ijms18010048

**Published:** 2016-12-28

**Authors:** Fa Yang, Weihong Wen, Weijun Qin

**Affiliations:** 1 Department of Urology, Xijing Hospital, Fourth Military Medical University, 127 Changle West Road, Xi’an 710032, China; yangfa@fmmu.edu.cn; 2 Department of Immunology, Fourth Military Medical University, 169 Changle West Road, Xi’an 710032, China

**Keywords:** bispecific antibodies, immunotherapy, immunological synapse, bispecific T cell engagers (BiTEs), angiogenesis inhibitors, delivery system, drug resistance

## Abstract

With the development of molecular cloning technology and the deep understanding of antibody engineering, there are diverse bispecific antibody formats from which to choose to pursue the optimal biological activity and clinical purpose. The single-chain-based bispecific antibodies usually bridge tumor cells with immune cells and form an immunological synapse because of their relatively small size. Bispecific antibodies in the IgG format include asymmetric bispecific antibodies and homodimerized bispecific antibodies, all of which have an extended blood half-life and their own crystalline fragment (Fc)-mediated functions. Besides retargeting effector cells to the site of cancer, new applications were established for bispecific antibodies. Bispecific antibodies that can simultaneously bind to cell surface antigens and payloads are a very ideal delivery system for therapeutic use. Bispecific antibodies that can inhibit two correlated signaling molecules at the same time can be developed to overcome inherent or acquired resistance and to be more efficient angiogenesis inhibitors. Bispecific antibodies can also be used to treat hemophilia A by mimicking the function of factor VIII. Bispecific antibodies also have broad application prospects in bone disorders and infections and diseases of the central nervous system. The latest developments of the formats and application of bispecific antibodies will be reviewed. Furthermore, the challenges and perspectives are summarized in this review.

## 1. Introduction

An increasing number of monoclonal antibodies have obtained approval and entered clinical applications [1]. However, despite the success, the limitations of antibody therapy are also not negligible. For example, the majority of patients who initially respond to monoclonal antibody treatment, which works through blocking signaling pathways and the induction of apoptosis, eventually relapse due to extensive cross-talk among some signaling pathways [2]. Antibody-dependent cell-mediated cytotoxicity (ADCC) and complement-dependent cytotoxicity (CDC) are the major effector functions of many therapeutic antibodies, while these effects vary for different antibodies, and the treatment effectiveness is not certain. Thus, in an attempt to overcome the shortcomings of monoclonal antibody therapy and improve the treatment effectiveness, monoclonal antibody-based bispecific antibodies have been raised. The concept of constructing bispecific antibodies as a therapeutic strategy was conceived of more than 20 years ago [3,4,5,6]. The key point of bispecific antibodies is that they can bind two antigens simultaneously. This leads to a wide concept for the construction of diverse bispecific antibodies, and thus, a large number of technologies for constructing bispecific antibodies have emerged. Quadroma technology, knobs-into-holes technology, CrossMAb technology, molecular cloning technology and the applications of linkers of different lengths are all extensively used. As a result, two bispecific antibodies have acquired approval with a large number of bispecific antibodies entering clinical trials. Furthermore, the application of bispecific antibodies is extended from the traditional tumor immunotherapy to many other diseases, like infections, acquired immune deficiency syndrome (AIDS) and genetic disease.

## 2. Diverse Formats of Bispecific Antibodies Meet the Optimal Biological Activity and Clinical Purpose

With the evolving of technologies that have enabled the engineering of recombinant protein derivatives, many different bispecific antibodies have emerged as therapeutic proteins. There are mainly two formats of bispecific antibodies, which are the single-chain-based format and the IgG-based format.

### 2.1. Single-Chain-Based Formats

**Figure 1:**
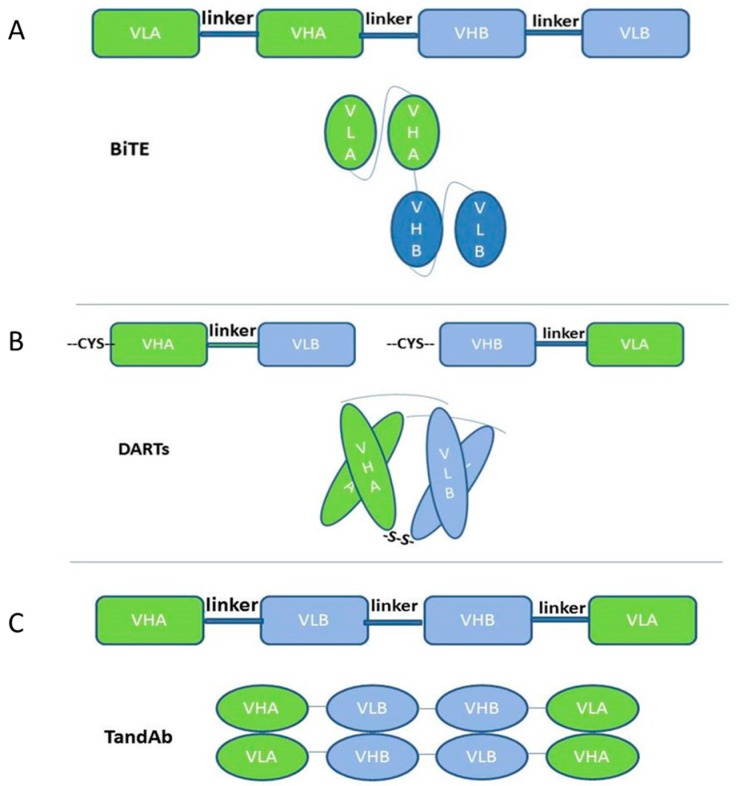
Single-chain-based formats. (**A**) The structure of BiTEs. The two linkers outside the chain are about 15 amino acids (G4S1)3, which is sufficiently long and flexible to make light chains and heavy chains form the right conformation; (**B**) The structure of dual-affinity re-targeting proteins (DARTs). The linker between heavy and light chains is as short as about five amino acids. Because of the short linker between the two domains of scFv, the two domains of the same scFv cannot pair and are forced to homodimerize with its homologous partner in another scFv. The adding of another cysteine residue at the end of VHA and VHB is helpful for the stability of this kind of bispecific antibody by forming a disulfide linkage; (**C**) The structure of Tandem diabodies (TandAbs). The three linkers of TandAb are all short linkers; it is hard to pair within the chain while easy to dimerize between two chains and generate.

By using molecular cloning technology, respective cDNAs encoding the variable domains of each parental monoclonal antibody and the linkers are cloned and linked together to form a single-chain bispecific antibody [7]. Two or more tandem repeats of different scFv fragments are cross-linked by a linker. The length of the linker is about 5–15 amino acids and Gly-Ser enriched. The most frequently-used domain order is VLA-linker1-VHA-linker2-VHB-linker3-VLB (VL and VH derive from the single chain antibody fragment, scFv; A and B represent the parental monoclonal antibody A and B), and the most frequently-used linker is GGGGS (G4S1, G and S represent respectively the amino acid Gly and Ser) repeats, which are non-immunogenic [8]. The length of the linker1 and linker3 determines the polymerization situation of scFvs, while the linker2 determines the movement flexibility between two scFvs. The linkers optimize the ability of scFvs to bind both the target antigens by regulating the number of repeats contained. Like one of the best known BiTEs blinatumomab, two longer linker sequences are placed between the heavy chain and the light chain to form the correct scFvs. A short linker sequence is used to bridge the two scFvs in tandem [9]. The longer linker is about 15 amino acids (G4S1)3, which is sufficiently long and flexible to make light chains and heavy chains form the right conformation (Figure 1A). However, the long length of the linker is also leading to the undesired pairing of the VH and VL domains that should not have been paired, thus leading to the dimeric forms besides the monomeric forms, while only the monomeric form is being pursued for its superior biological and pharmaceutical properties [10].

While for the dual-affinity re-targeting proteins (DARTs), the linker between heavy and light chains is as short as about five amino acids, the most frequently-used domain order for DARTs is VHA-linker-VLB, VHB-linker-VLA. Because of the short linker between the two domains of scFv, the two domains of the same scFv cannot pair and are forced to homodimerize with its homologous partner in another scFv, which means that VHA cannot pair with VLB, and it has to pair with VLA [11]. The adding of another cysteine residue at the end of VHA and VHB is helpful for the stability of this kind of bispecific antibody by forming a disulfide linkage (Figure 1B). If the domain order is VHA-outer linker-VLB-central linker-VHB-outer linker-VLA and both outer linkers are comprised of six amino acids (Gly-Gly-Ser-Gly-Gly-Ser), while the central peptide linker was one of three sequences: Gly-Gly-Ser-Gly, Gly-Gly-Ser-Gly-Gly or Gly-Gly-Ser-Gly-Gly-Ser, it is easy to dimerize and generate a new kind of bispecific antibody: Tandem diabodies (TandAbs) [12,13] (Figure 1C). TandAbs have a molecular weight (105 kDa) exceeding the renal clearance threshold, thus offering a longer half-life compared to smaller antibody constructs, like BiTEs [13]. It would exhibit high potency because the format of TandAbs is bivalent for each specific binding; while it also permits a similarly close distance of two antigens, like BiTEs do [14].

These single-chain-based molecules usually target tumor-associated antigens with one antigen binding site and target leukocyte antigens with another to recruit immune cells [15]. The advantage of such molecules is the reduced size that probably results in enhanced tissue penetration. Furthermore, these kinds of molecules are easily bridging tumor cells and immune cells to create an immune synapse due to its short arm [16,17]. In the case of the BiTEs, the immunological synapses formed by BiTEs are nearly indistinguishable from those formed in the course of natural cytotoxic T cell recognition [16]. As a result, the crosslinking mediated by these single-chain bispecific antibodies results in the effective recruiting of lymphocytes to tumor targets and the subsequent release of cytotoxins into the milieu of the immunological synapse. These artificially-recruited cytotoxic lymphocyte (CTLs) can bypass the MHC context required for antigen recognition by the T cell receptors (TCR) [18]. However, the short in vivo half-life is their weakness due to the absence of the Fc-located binding site of the neonatal Fc receptor FcRn. Additionally, because of their relatively small construct size (especially for BiTEs of only 55 kDa), they are easily excreted by the kidneys. Thus, they require prolonged continuous intravenous administration [19]. Besides being constructed on the Fc structure, the half-life of these small molecules can also be prolonged by adding some modified proteins, like modified human serum albumin [20].

### 2.2. IgG-Based Formats

The Fc of IgG is a very attractive scaffold for designing novel therapeutics because it contains all antibody functions except the binding ability. Fc engineering is important for improving the effectiveness of the bispecific antibodies. For example, the aim of prolonging the half-life, enhancing or reducing the ADCC or CDC effect and mutating the Fc domain to realize the heterodimerization can be realized by Fc engineering [21]. Hence, it is necessary to illuminate the structure of Fc clearly. Most antibody-based therapies use IgG as the scaffold to construct their entities. Here, we will focus on the structure of IgG Fc [22]. A full-sized IgG antibody can be dissected into two parts: Fab and Fc. Fc is a homodimeric glycoprotein. Each of the two chains consists of a hinge region, the CH2 and CH3 domains. The hinge region links the Fab and Fc and forms interchain disulfide bonds between the two heavy chains [23]. CH2 of each heavy chain is attached with a *N*-linked glycan, which is critical for the structural stabilization of the antibody [24]. There is an extensive interaction between CH3 domains. Additionally, the interaction between CH3 domains is the key factor that promotes the assembly of two heavy chains and the formation of two interchain disulfide bonds, which is the base of engineering the CH3 domain to realize the concept of some technologies, like knobs-into-holes technology (described in detail below).

**Figure 2:**
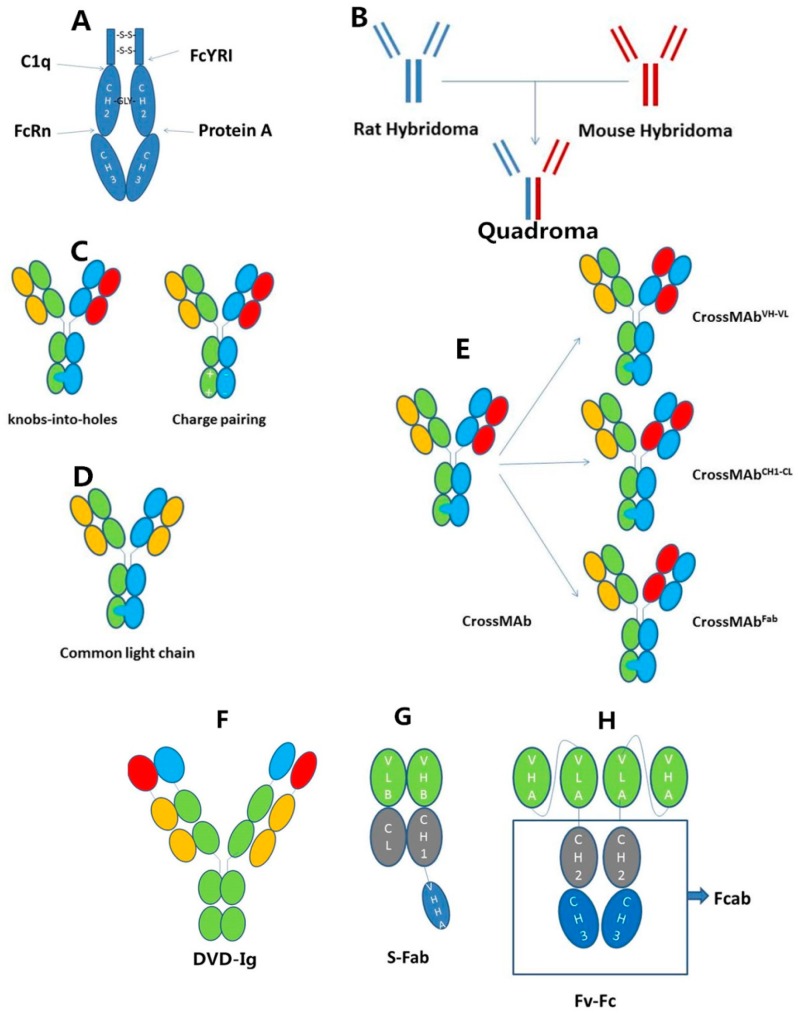
IgG-based formats. (**A**) The structure of Fc and its binding ligands. Dark arrows mark the binding site of different binding ligands; (**B**) The formation of quadroma cells to produce rat/mouse bispecific antibodies; (**C**) “Knobs-an-holes” and charge pairing technology solving the heavy chain mispairing problem; (**D**) The common light chain technology solving the light chain mispairing problem; (**E**) The three formats of crossmab, the exchange of VHB and VLB, the exchange of CH1 and CLB and the exchange of VHB-CH1 and VLB-CLB; (**F**) The format of double-variable domain (DVD)-Ig; (**G**) The format of S-Fab; (**H**) The format of Fv-Fc.

Except for antigen binding, the other functions of IgG are all associated with the Fc fragment (Figure 2A). One of the most important functions is antibody-dependent cell-mediated cytotoxicity (ADCC), which is mediated by FcγR. FcγR is expressed on myeloid cell lines, especially the natural killer (NK) cells. These effector cells are activated by binding with the Fc domain of antibodies, which have already been binding with antigens, and release cytotoxins like perforin and granzymes to induce the apoptosis of target cells, like tumor cells. The binding site of FcγR is at the lower hinge region and the proximal region of the CH2 domain. Similar to FcγRs, the binding site of complement C1q is also at the center of the hinge and CH2 domain. The binding of IgG-Fc and C1q results in the effect of complement-dependent cytotoxicity (CDC). Another important receptor of IgG-Fc is FcRn [25]. FcRn is expressed mainly at vascular endothelium and protects IgG from degradation. The binding of IgG-Fc and FcRn is sensitive to the pH value (a numeric scale used to specify the acidity or basicity of an aqueous solution). As endothelial cells internalize serum proteins, the FcRn-IgG-Fc compound is also internalized, but then, IgG is released and returns to the circulation due to the low affinity of binding to FcRn in the endosome, in which the pH is different from blood. Due to this mechanism, the half-life of IgG is prolonged by about 6–8 days; while without FcRn, it only sustains one day. The binding site of IgG-Fc for FcRn is at the CH2–CH3 inter-domain region [26]. This region is also the binding site for protein A, a bacteria protein produced by *Staphylococcus aureus*. Protein A is now extensively used for the purification of mAb formats and recombinant proteins containing the Fc fragment [27].

The structure of Fc and the structure-based function are the foundations for the design of bispecific antibodies containing the Fc unit. The CH3 domain is a potential site for researchers to modify and add some entities to create multifunctional molecules.

The idea to combine the halves of two different Y-shaped antibody molecules seems promising, but there are problems with producing the desired bispecific antibody. Because normally, all heavy chains can pair with each other and every light chain can bind unspecifically to two regions at the top of the heavy chains, these problems are the heavy (H) chain mispairing problems and light (L) chain mispairing problems. Bispecific antibodies in the IgG format have traditionally been generated by the fusion of two different hybridoma cells, which resulted in a “quadroma” cell line (Figure 2B). The rat/mouse quadroma technology can solve the heavy chain mispairing problems and light chain mispairing problems. These bispecific antibodies consist of two half antibodies that originate from parental mouse IgG2a and rat IgG2b hybridomas. Each of these half antibodies has one light chain and one heavy chain. In rat/mouse quadromas, the H chains are preferentially associated with L chains from the same species; therefore, the L chain of mouse IgG2a will pair with the H chain of mouse IgG2a; likewise, the L chain of rat IgG2b will pair with the H chain of rat IgG2b. Furthermore, because rat antibodies cannot bind to protein A, but mouse antibodies have a high affinity to bind protein A, undesired homologous rat H chain pairing and the contaminating bovine immune globulins are removed when the quadroma products flow through the protein A purification column. While undesired homologous mouse H chain pairing is tightly bound to protein A by their two sites of two mouse H chains, only rat/mouse bispecific antibodies are moderately bound to protein A. Thus, rat/mouse bispecific antibodies can then be easily eluted at a near-physiologic pH of 5.8, whereas parental mouse antibodies are stably retained in the protein A column. Therefore, through the purification by protein A, the H chain mispairing problem is also solved [6]. However, the rat-mouse hybrid format introduces nonhuman sequences, which may result in immunogenic responses that accelerate clearance and inhibit the function of the antibody in humans.

“Knobs-into-holes” technology is the point mutation within the CH3 domain of each heavy chain. Knobs are constructed by replacing small side residues with the large side residues. Holes of an identical or similar size to the knobs are created by replacing large side residues with the smaller ones. Finally, Knob inserts into an appropriately sizeable hole on the partner CH3 domain. In this way, the H chain of parental monoclonal antibodies that are expressed in the same cell can correctly pair and assemble into the bispecific antibodies (Figure 2C) [5]. Another way to create heterodimers of bispecific antibodies is also the selected mutation of the CH3 domain of each heavy chain. These novel mutations create altered charge polarity across the interface region of Fc. For example, the Fc of IgG A is positively charged, and the Fc of IgG B is negatively charged, so that the half of IgG A and the another half of IgG B preferentially form heterodimers through favorable attractive interactions, while unfavorable repulsive charge interactions suppress unwanted Fc homodimer formation (Figure 2C) [28].

These two ways enable correct heavy chain association, but they cannot prevent the undesired pairing of the light chains associated with the two different heavy chains, because the molecular architectures of VH and VL, CH1 and CL (CH1 represents the first constant domain of the antibody heavy chain. CL represents the constant domain of the antibody light chain) domains in both arms of a bispecific antibody are identical.

There are many ways to solve this problem. One way is to express antibodies with knobs and antibodies with holes respectively in different cells and to mix them later, so that the corresponding pairing of light chain and heavy chain has already been completed before being mixed [29]. This improved technology produces a high rate of heterodimers with the right pairing of the corresponding light chains. Another way is the “common light chain” approach (Figure 2D). Some antibodies that bind to different antigens share identical light chains. These antibodies were engineered to form bispecific antibodies without worrying about the mispairing of light chains [3]. However, this way is restricted because it is uncommon to find the common light chains that recognize different antigens.

The most extensively-used method is the CrossMAb technology (Figure 2E). CrossMAb technology is a generic approach that solves the light chain mispairing problems by exchanging heavy chain and light chain domains within the antigen binding fragment (Fab) of one half of the bispecific antibody. CrossMAbs have no chemical linkers nor connectors and prevent binding of the light chains to its heavy counterparts to prevent unwanted side products. This “crossover” still retains the original antigen-binding affinity, but makes the two arms so different that only the specific interaction can take place. There are three ways for this change: the exchange of VH B and VL B, the exchange of CH1 and CL B and the exchange of VH B-CH1 and VL B-CL B, all of which occur at the gene level [30].

Besides asymmetric bispecific antibodies, homodimerized bispecific antibodies have been getting increasing attention. A single-chain Fv fragment or a domain antibody can be added to the N or C terminus of the heavy or light chains, respectively, of an antibody by a flexible linker, resulting in tetravalent symmetrical IgG-like proteins with two binding sites for each antigen. Double-variable domain (DVD)-Igs belong to this class of symmetric bispecific IgG and IgG-like molecules. DVD-Igs consist of four additional variable domains (VD) connected by a linker to the N termini of heavy and light chains of IgG molecules (Figure 2F) [31]. The procedure of manufacturing and purification and the pharmacological properties of (DVD)-Igs are similar to those of a conventional IgG1. The affinity and function of the second variable domain can be tuned by adjusting the length and changing the sequence of the linkers between two variable domains [32].

Single-domain antibodies are also known as nanobodies. These heavy chain-only antibodies are antigen specific, have high binding affinity and can be further truncated to produce isolated variable domains (VHH, variable domain from heavy-chain-only antibodies) without significant loss of their antigen-recognizing properties [33]. Fab and single-domain antibody VHH are used in the construction of bispecific antibodies. The VHHA is cloned into the C terminal of VHB-CH1, and then, the heterodimerization of VHB-CH1-VHHA and VLB-CL forms the bispecific antibody S-Fab (Figure 2G) [34,35].

Fcab is a mutant Fc fragment with specificity against another antigen [36]. Changing the base sequence of some special sites of CH3 domain does not affect the function and structure of the antibody because most of the functions are performed by the hinge and CH2 domains. Thus, this theory provides a chance to remold the Fc fragment and find antigen-specific Fc binders, named Fcab, which bind antigens through the C-terminal tip of Fc [37]. A new emerging bispecific antibody is the bispecific Fv-Fc antibody, in which a single chain variable fragment for one antigen is fused to the C terminus of Fcab (Figure 2H). The size of the bispecific Fv-Fc antibody is similar to the normal IgG antibodies, but with two specificities.

Bispecific antibodies with an Fc region have an extended blood half-life and own Fc-mediated functions. However, if the aim is only to prolong the half-life, yet bypass the other effects, it can be met by engineering and mutating some specific sites to ban the effects of the activation of innate effector cells [38].

## 3. The Mechanism of Action for Bispecific Antibodies Is Like a Bridge Linking Two Cells or Blocking Two Antigens in the Same Cell

Bispecific antibodies have special mechanisms of action, including two most distinct and promising aspects: (1) as a bridge to link target cells with effector cells or drug moieties; (2) binding two epitopes on the same cell to simultaneously block two compensatory signaling pathways or to improve affinity and avoid side effects. The first aspect will be discussed in the following section. This section is to discuss the second aspect.

Bispecific antibodies that are capable of interacting with two different epitopes on the same antigen [39] or different antigens on the same cell [40] can induce receptor cross-linking and result in high affinity. The bispecific antibodies that target two different epitopes on HER2 cross-link HER2 and form a larger meshwork structure than a monoclonal antibody does [23,39]. Additionally, because of the surface diffusion, the distance of different antigens that previously exceeded the reach of the antigen-binding arms of the antibody can be pulled near each other by bispecific antibodies, which allows for the highly specific binding of bispecific antibodies to antigens on the same cell, despite these two antigens’ surface density being too low [40]. These bispecific antibodies form a trimeric complex with the targeted antigens on the same cell [20]. This demonstration can shed light on more novel drug discoveries. Anti-CTLA-4 (cytotoxic T-lymphocyte-associated protein 4) and anti-PD-1 (programmed cell death protein 1) monoclonal antibodies were approved by the U.S. FDA for the treatment of melanoma and are undergoing clinical trials for the treatment of many other tumors. Though these checkpoint inhibitors have revolutionized the treatment of advanced cancers, they also results in a spectrum of adverse events [41]. A bispecific antibody that consists of both of these two specificities may lead to a higher specificity and cause less adverse events.

Next, the progress and application of bispecific antibodies will be introduced in detail through the following aspects respectively: (1) retargeting cellular immunity towards the malignant cells; (2) delivering cytotoxic entities to the malignant cells; (3) modifying the host response; (4) inhibiting tumor angiogenesis; (5) directly targeting the malignant cells; and (6) treating other diseases besides tumors. (1)–(5) mainly focus on the treatment of tumors, while (6) introduces the bispecific antibodies applied innovatively for other diseases besides tumors. Table 1 lists some representative bispecific antibodies that are entering clinical trials or already entering the market.

### 3.1. Retargeting Cellular Immunity towards the Malignant Cells

These kinds of bispecific antibodies recruit effector cells from the immune system of the host, for the selective destruction of cells, especially tumor cells expressing the targeted antigens. One of their antibody moieties identifies the tumor cells via a tumor-specific antigen, while the second antibody binding site is used to recruit and activate a suitable leukocyte. T cells and NK cells are the main factors that help to kill tumor cells; therefore, T cells and NK cells are the most frequently recruited cells by bispecific antibodies [42,43]. Bispecific T cell engagers, termed BiTEs, with one scFv binding to a T cell-specific molecule, usually CD3, while the second scFv binds to a tumor-associated antigen, efficiently enhance the patient’s immune response to tumors. The T cells are activated and release cytokines to kill tumor cells when the synapse is formed between T cells and the tumor cells.

Blinatumomab is a murine-derived bispecific T cell engager (BiTE) antibody that directs CD3-positive T cells against CD19-positive B cells. It works by inducing the proliferation and the activation of T cells to exert cytotoxic activity on the malignant cells [8]. Due to its excellent effect in clinical trials, blinatumomab received an accelerated approval from the FDA for the treatment of Philadelphia chromosome-negative relapsed or refractory acute lymphoblastic leukemia in December 2014 [44]. It is currently investigated in pediatric ALL, because CD3 and CD19 are positive in pediatric, as well as adult patients [45]. Blinatumomab might also benefit patients with non-Hodgkin’s lymphoma, especially for those with aggressive forms, like diffuse large B cell lymphoma [46]. Another three BiTEs are also entering clinical trials: anti-EpCAM/CD3 MT-110 [47], anti-CEA (carcino-embryonic antigen)/CD3 MT-111 [48] and anti-PSMA (prostate specific membrane antigen)/CD3 AMG 212 [50]. All of these BiTEs have already shown high antitumor activity in diverse xenograft models and were tested in the corresponding clinical trials. A study has also shown the capacity of MT-110 to eradicate cancer stem cells [59].

DART proteins are the heterodimer of two peptides derived from two parent antibodies. Because DART protein is larger in size than BiTEs, it shows extended storage and serum stability. Functionally, the DART proteins have demonstrated extremely potent, dose-dependent cytotoxicity in redirecting human PBMC against targeted cell lines [60]. MGD006 is a CD3 × CD123 DART molecule that induces T cell target-specific association, activation and proliferation. The high stability and excellent performance of DARTs enhanced the clinical application [51]. As a result, it has entered the phase I clinical trial in relapsed/refractory acute myeloid leukemia (AML) or intermediate-2/high risk myelodysplastic syndromes (MDS) (ClinicalTrials.gov: NCT02152956).

Stadler et al. engineered a T cell-engaging bispecific single chain molecule (bi-(scFv)_2_) with anti-CD3 and anti-CLDN6 specificities, with its format similar to BITEs. CLDN6 is the fetal tight junction molecule claudin, highly expressed in various solid tumors, whereas barely expressed in normal tissues. This kind of bispecific antibody also showed the efficacy of recruiting T cells and eradication of tumors in xenograft models [61].

The bispecific NK cell engager (BiKE) is also constructed to recruit NK cells. Aifen Li et al. constructed a HER2-S-Fab with a binding site for CD16 to recruit NK cells to kill HER2-positive tumor cells [34]. Schmohl et al. constructed another NK cell engager by incorporating IL-15 between the anti-EpCAM region and the anti-CD16 region. This new form showed the ability of both facilitating the ADCC effect and the induction of NK cell expansion [62]. NKG2D is an activating receptor found on NK and CD8^+^ T cells. Its ligand ULBP2 and MICA are attached through linkers to the scFvs, which target tumor antigens, like CD138, CD19, CD33 and CD24, to generate kinds of bispecific antibodies. A recombinant bispecific protein (ULBP2-BB4) binds the activating NK receptor NKG2D and the CD138, which is overexpressed on a variety of malignancies [63]. A mono-targeting triple body ULBP2-aCD19-aCD19 and a dual-targeting triple body ULBP2-aCD19-aCD33 were constructed to recruit NK cells to kill CD19- or CD33-positive chronic lymphocytic leukemia cells [64]. MICA was attached to rG7S, a single-chain antibody fragment (scFv) targeting the tumor-associated antigen CD24 to form a fusion protein rG7S-MICA to treat hepatocellular carcinoma (HCC) [65]. All of these four recombinant proteins can strongly activate primary NK cells in vitro and in vivo and enhance the NK cell-mediated lysis of corresponding antigen-positive tumor cells [63,64,65].

Because of offering a longer half-life compared to smaller antibody constructs, TandAbs are a hotspot to be developed. Unlike the BiTEs, TandAb has two binding sites for one antigen and another two binding sites for the second antigen. TandAb mediates the killing of tumor cells with little dependence on the potency or efficacy upon the effector:target ratio, and it can effectively kill tumor cells at lower effector: target ratios [3,14]. Thus, some promising TandAbs are coming to the fore, like CD33/CD3 TandAb [13], CD19/CD3 AFM11[14] and CD30/CD16A AFM13 [52]. AFM11 induced dose-dependent growth inhibition and exhibited substantial cytotoxic activity both in NOD/SCID xenograft models and in vitro. Because of its excellent preclinical outcomes, AFM11 is entering clinical trials and currently recruiting patients with relapsed and/or refractory CD19-positive B cell non-Hodgkin lymphoma (NHL) (ClinicalTrials.gov: NCT02106091).

AFM13 is designed for the treatment of CD30-expressing malignant lymphomas by recruiting and activating natural killer (NK) cells via binding to CD16A. Preclinical data demonstrate a specific and efficient antitumor activity via the engagement of NK cells [12]. In the phase I study, the treatment with AFM13 was well tolerated, and the treatment caused recruitment and activation of NK cells [52]. It is now entering phase II clinical trials (ClinicalTrials.gov: NCT02321592).

Catumaxomab (anti-EpCAM/anti-CD3) is produced by a quadroma cell line. The quadroma cell line for the production of catumaxomab is obtained by the fusion of two parental hybridoma cell lines: a mouse hybridoma cell line expressing anti-human EpCAM antibody and a rat hybridoma cell line producing anti-human CD3 antibody. Catumaxomab has the ability to bind three different cell types: EpCAM positive tumor cells, T cells and accessory cells via its intact Fc region. It was approved in the European Union on 20 April 2009 for the treatment of malignant ascites (MA), a condition occurring in patients with EpCAM-positive metastasizing cancer where standard therapy is no longer feasible [53]. It is safe and effective for catumaxomab to be given both to hospitalized patients and outpatients [54]. Toxicity was generally manageable, with abdominal pain, nausea/vomiting, fatigue and fevers the predominant adverse effects; while its trifunction also causes some side effects, like liver injury. Catumaxomab binds to FcγR-positive Kupffer cells to stimulate the release of C-reactive protein, chemokines and cytokines, which induces liver injury [66]. Besides catumaxomab, there are other similar bispecific antibodies composed of an anti-CD3 rat IgG2b half antibody for T cell recognition and the antigen binding site presented by the mouse IgG2a isotype: HER2/CD3 ertumaxomab for breast cancer treatment [55] and CD20/CD3 FBTA05 for pediatric high-risk patients with recurrent CD20-positive B cell malignancies [56]. The phase II clinical trial of ertumaxomab has terminated (clinicaltrials.gov: NCT00351858). FBTA05 is being tested in a phase I/II clinical trial evaluating the safety and efficacy of FBTA05 in combination with donor lymphocyte infusions for the treatment of relapsed or refractory disease in CD20-positive non-Hodgkin lymphoma after allogeneic transplantation (clinicaltrials.gov: NCT01138579).

CrossMab and knobs-into-holes technology are also widely used to overcome the obstacle of the light chain mispairing problem. CEA TCB is the IgG1-based bispecific heterodimeric antibody that binds with one arm to CD3 expressed on T cells and with two arms to CEA expressed on tumor cells. It adopted the crossmab and knobs-into-holes technology to make sure that the H and light chains pair correctly. The correct association of light chains of the antibody is enabled by introducing a CH1-CL crossover into the internal CD3-binding Fab. It also incorporated the engineered Fc region with completely abolished binding to Fcγ Rs and C1q [17,38]. CEA TCB is now entering clinical trials and getting promising results (ClinicalTrials.gov: NCT02324257).

However, more notably, the recruited T cells may be severely hampered by the dysfunction of infiltrated pre-existing T cells, as they may present with a high frequency of inhibitory checkpoint molecules like PD-1, thus resulting in significantly impaired tumor cell killing of recruited T cells. Jens Schreiner et al. characterized inhibitory receptors of pre-exiting T cells and found that highly expressed PD-1 infiltrated T cells defined a T cell subset with particularly high levels of multiple inhibitory receptors. Blocking PD-1 could restore the cytokine secretion of infiltrated T cells [67]. Thomas Köhnke et al. also presented in a report a case of a 32-year-old male patient with refractory B-precursor ALL who was resistant to treatment with blinatumomab. Bone marrow immunohistochemistry showed the T cell infiltration and the increasing in the expression of PD-L1 as a potential immune escape mechanism [68]. As the bispecific antibody can efficiently activate T cells, resulting in the production and release of a large number of inflammatory cytokines, bispecific antibody therapy may also trigger immune evasion of antibody-mediated tumor cell lysis of tumor cells. Christina Krupka et al. found that although the expression of PD-1 and PD-L1 was extremely low in patients with primary AML, their expression could be induced by AMG 330-mediated (a bispecific CD33/CD3 BiTE antibody construct) T cell activation. Additionally, blockade of the PD-1/PD-L1 interaction resensitizes target cells to AMG 330-mediated lysis [69]. Regulatory T cells (Tregs) interfere with T cell function, leading to the failure to produce strong cellular and humoral immune responses that can affect the efficacy of recruited T cells. One clinical trial’s data suggest that cytotoxic T lymphocyte-associated antigen-4 (CTLA-4)-positive Tregs are associated with diminished T cell antitumor activity [70]. Yano et al. used ipilimumab, an antagonistic monoclonal antibody against CTLA-4, to enhance bispecific antibody (BiAb)-redirected antitumor cytotoxicity of activated T cells by inhibiting the immunosuppressive activity of regulatory T cells (Tregs). They found that the presence of ipilimumab not only enhanced the amplification of activated T cells, but also enhanced the secretion of cytokines and the specific cytotoxicity of T cells [71].

The method of adoptive T cell therapy combined with bispecific antibodies can be applied to address these limitations. Recent studies have shown that activated T cells armed with bispecific antibodies have promising efficacy in clinical trials and can be adapted to restore the sensitivity toward bispecific antibodies aiming to recruit immune cells [72]. Briefly, activated T cells were produced by activating PBMC with soluble anti-CD3 monoclonal antibodies and expanded by adding IL-2 for 14 days. After culturing, activated T cells were harvested, washed, counted and resuspended, and then, they were armed with bispecific antibodies at different ratios for one hour at 4 °C. These T cells are derived from normal donors or cancer patients [73]. Arming in vitro expanded T cells with bispecific antibodies may not only improve clinical responses by bypassing the depression of the tumor microenvironment, but also minimize toxicity by avoiding the cytokine storm that can occur by systemic infusion of bispecific antibodies alone [74]. A phase I clinical trial was conducted, which included 23 women with metastatic breast cancer. Each one received the administration consisting of eight infusions of anti-CD3 × anti-HER2 bispecific antibody (HER2Bi) armed with anti-CD3 activated T cells (ATC) in combination with low dose interleukin 2 (IL-2) and granulocyte macrophage colony stimulating factor (ClinicalTrials.gov: NCT01147016). The results showed that infusions of activated T cells armed with bispecific antibodies induced anti-tumor responses, and the Th1 cytokines and IL-12 serum levels increased [74].

Katarzyna Urbanska et al. also designed a combination platform to overcome these shortcomings. This platform includes unique bispecific antibodies and engineered T cells. Bispecific antibodies are bound with folate receptors and tumor-associated antigens. T cells were engineered to express a novel chimeric receptor comprised of an extracellular folate receptor fused to intracellular TCR and CD28 costimulatory signaling domains. In this way, bispecific antibodies can stimulate the engineered T cells to kill tumor cells and avoid the influence of the suppressive tumor environment [75]. An article published recently pointed out another way to integrate bispecific antibody into T cells. T cells were engineered with synthetic Notch (synNotch) receptors, which can induce transcriptional activation, and with the genes that express bispecific antibodies, like BiTEs. As a result, when T cells were activated by one antigen expressed on tumor cells, the synNotch signaling will induce the expression and secretion of BiTEs; BiTEs will then activate T cells in turn [76].

### 3.2. Delivering Cytotoxic Entities to the Malignant Cells

Bispecific antibodies that can simultaneously bind to cell surface antigens and payloads are a very ideal delivery system for therapeutic use. Digoxigenin (Dig), as a hapten, can be used as the payload scaffold to embark on some cytotoxic moieties, such as fluorophores, chelating agents, chemotherapeutics, nucleic acids, lipids, nanoparticles or peptides and proteins, and finally, form compounds, like Dig-Cy5, Dig-doxorubicin and Dig-GFP [77]. Bispecific antibodies that target the cell membrane antigens and digoxin (DIG) were produced for targeted payload delivery. Targeting antibodies can bind the tumor antigens, such as HER2. A Dig-binding single-chain Fv was fused to the C terminus of the CH3 domains of targeting antibodies in a disulfide-stabilized form. Because Dig-bispecific antibodies can also effectively capture digoxigeninylated compounds under physiological conditions, separate administration of uncharged Dig bispecific antibodies followed by application of Dig payload is sufficient to achieve antibody-mediated targeting in vitro and in vivo [78].

Delivering siRNA into tumor cells and knocking down some vital genes is also a promising method for cancer therapy. Hapten-based bispecific antibody is also an effective siRNA delivery system [79]. siRNA was digoxigeninylated at its 3′-end and formulated into nanoparticles consisting of dynamic polyconjugates (DPCs) or into lipid-based nanoparticles (LNPs). This Dig-siRNA was bound in a 2:1 ratio to the bispecific antibodies, which bind tumor antigens, such as HER2, IGF1-R, CD22 and LeY. These bispecific antibody-siRNA complexes delivered siRNAs specifically to cells expressing the corresponding antigen, then the complexes were internalized into endosomes, and Dig-siRNAs were separated from bispecific antibodies. An in vitro study showed that the complexes induced the knockdown of targeting mRNA by specific siRNAs for a variety of bsAbs, siRNAs and target cells. An in vivo study in mice bearing tumor xenografts showed that CD31 mRNA was significantly knocked down in endothelial cells after systemic co-administration of bispecific antibodies and LNPs containing siRNAs that were targeted to the CD31 of tumor vasculature [80].

Diphtheria toxin (DT) mediates potent cell-cycle-independent cell death and, therefore, can be particularly effective as an alternative therapy for chemotherapy refractory malignancies. Vallera et al. fused bispecific scFvs targeting human CD19 and CD22 cell surface receptors with DT as a new therapy against CD22^+^CD19^+^ systemic B cell malignancy. This new agent and its variant version DT2219ARL resulted in long-term tumor-free survivors measured in a bioluminescent xenograft imaging model [57]. Because of the great anti-cancer activity, DT2219 entered phase 1 clinical trial and showed safety and effectivity [58] (ClinicalTrials.gov:NCT02370160, NCT00889408).

As radio immunotherapy (RIT) enables the delivery of tumoricidal radiation doses to multiple systemically-dispersed sites simultaneously, it is feasible for diseases that have already metastasized to multiple organs. For this purpose, bispecific antibodies that target tumor antigens, as well as compounds containing radioactive materials were generated. A single-chain antibody fragment against DOTA complexed with β particle-emitting radio metals, such as 177Lu and 90Y, was attached to a full humanized monoclonal antibody targeting a tumor antigen GD2 [81]. In the same way, another bispecific antibody with dual specificity for GPA33 tumor antigen and the DOTA-Bn (radio lanthanide metal) complex was tested [82]. All of these radio immunotherapy agents can effectively bring radioactive materials into the tumor site and promisingly ablate corresponding tumors in mice, while sparing kidney and bone marrow accumulation.

Bacterial minicells are anucleate nanoparticles produced as a result of inactivating the genes that control normal bacterial cell division, thereby derepressing polar sites of cell fission. The minicells are purified through the use of a process to remove pollutants, such as parent bacterial cells, cell debris, cellular components, free nucleic acids and free endotoxin. Then, chemotherapeutic drugs were packaged into minicells. Finally, minicells loaded with chemotherapeutic drugs were targeted via bispecific antibodies to antigens on the membrane of cancer cells, which results in endocytosis, intracellular degradation and drug release. This new delivery system generates a significant tumor growth inhibition and regression in mouse xenografts [83]. Because of the great outcomes of preclinical studies, a phase I clinical study of bispecific antibody-mediated EGFR-targeted, paclitaxel-loaded minicells was initiated. This minicell delivery system was well tolerated and safe in patients and has shown modest clinical efficacy [84].

### 3.3. Modifying the Host Response against Drug Resistance

Activation of the extensive cross-talk among some signaling pathways and compensatory survival signals has emerged as a likely source of drug resistance. A high acquired resistance rate remains unsolved in patients treated with single drugs. Furthermore, inhibitory checkpoint molecules are the main inhibitory factors that hinder immunotherapy. Therefore, many therapeutic strategies have been actively developed to overcome inherent or acquired resistance. Bispecific antibodies are the best choice in that they can simultaneously inhibit two correlated signaling molecules.

Some studies have shown that the HER3 ligand, heregulin, is the main cause of drug resistance against PI3K inhibitor GDC-0941 by allowing prostate cancer cells to fend off the signaling pathway; while targeting HER2 and HER3 with corresponding antibodies restored the sensitivity of GDC-0941 and allowed the GDC-0941 to again stop the growth of the cancer. Thus, bispecific antibodies blocking HER2 and HER3 signaling may restore and enhance the effectiveness of PI3K inhibitors to treat prostate cancer in patients [85].

The immune checkpoint molecules are one of the main factors inhibiting adoptive immunotherapy. PD1/PDL1 and B7-H3 (CD276) play an important role in the inhibition of T cell function. High expression of B7-H3 predicts poor prognosis and poor clinical outcome [86]. Blocking B7-H3 can significantly inhibit tumor growth [87]. To prevent the negative effect of B7-H3 and activating T cells, the bispecific antibodies against B7-H3 and CD3 were constructed aiming to reshape the cytotoxic activity of T cells. When tested in vitro and in vivo, an increase in cytotoxic activity of B7-H3Bi-armed T cells against tumor cells was observed in contrast with unarmed T cells [88].

EGFR is overexpressed in certain types of human carcinomas especially in lung cancers and colorectal cancers. The overexpression causes the uncontrolled tumor cell proliferation. Thus, the blockade of EGFR by inhibitors like Gefitinib significantly inhibits the tumor cell proliferation; while despite the promising clinical efficacy of these inhibitors, most responders eventually obtained resistance. The amplification and activation of the Met oncogene have been reported as a chief culprit to be involved in acquired resistance to EGFR inhibitors [89]. In order to solve this problem, bispecific antibodies for Met/epidermal growth factor receptor (EGFR) and Met/HER2 were generated. These bispecific antibodies successfully induced an efficient EGFR or HER2 internalization and degradation in the presence of Met, offering a promising way to help restore the sensitivity to EGFR inhibitors [90].

### 3.4. Anti-Tumor Angiogenesis

Because of the promising antitumor effects in patients, bevacizumab, a monoclonal antibody that neutralizes VEGF to prevent new vascular formation, was approved for use in combination treatment with cytotoxic chemotherapy for metastatic colorectal cancer in 2004 [91]. However, the absence of tumor specificity by these agents frequently leads to off-target side effects. Accumulating evidence has demonstrated that the use of a single anti-angiogenic agent, the strategy most often used now, is unable to sufficiently inhibit tumor angiogenesis, leading to tumor recurrence and drug resistance; as tumor endothelial cells can still be stimulated by alternative angiogenic factors, like angiopoietin-2 (Ang-2) and Dll4, which are not blocked [92].

In preclinical and clinical studies, VEGF inhibitors alone have been shown to increase tumor hypoxia and promote tumor invasion and metastasis [93]. Hence, a new method must be put forward to meet these problems. A bispecific antibody (A2V) inhibiting Ang-2 and VEGF was constructed and evaluated in glioblastoma xenografts. In a tumor model with highly abnormal tumor vessels, A2V reduced vascular density, delayed tumor growth and prolonged survival compared with parental antibodies [94]. Another bispecific antibody HD105 against VEGF and Dll4 was developed. δ like ligand 4 (DLL4) is a promising antiangiogenic target because of its ability to expand the inhibitory effects of VEGF inhibitors on angiogenesis. The in vivo xenograft studies have shown that HD105 is more effective at inhibiting the progression of human lung cancer and gastric cancer than using anti-VEGF or anti-Dll4 antibodies alone [95].

### 3.5. Directly Targeting the Malignant Cells

For complex diseases, a variety of mediators contribute to the pathogenesis of the disease through different mechanisms, and inhibition of multiple targets may produce a better therapeutic effect than the blockade of a single target.

As mentioned above, the mechanism by which non-small cell lung cancer (NSCLCs) with activating EGFR mutations become resistant to tyrosine kinase inhibitors (TKIs) is the second mutation in EGFR and/or the activation of the C-met pathway. Additionally, these two resistant mechanisms are also compensatory when one pathway is blocked. Thus, blocking these two ways simultaneously will improve overall efficacy. Sheri L. Moores et al. introduced a bispecific antibody that targeted these two antigens and showed great results: blocking ligand-induced activation, inducing receptor degradation and, in addition, enhancing the ADCC effect [96].

Similar with EGFR and C-met, the activation of her3, the preferred dimerization partner of HER2, also plays a key role in driving HER2-amplified tumor growth. A bispecific antibody MM-111 was generated by the fusion of two different scFvs with modified human serum albumin. By binding to HER2 and HER3 and forming a trimeric complex, MM-111 effectively inhibited HER3 signaling and showed potential inhibition of the proliferation of HER2-expressing tumor cells in vitro and in vivo [20]. The phase I clinical trial of MM-111 has been completed, and the adverse events are rare and controllable (ClinicalTrials.gov: NCT00911898, NCT01304784).

### 3.6. Bispecific Antibodies Used Besides for Tumors

Emicizumab, a humanized bispecific antibody binding to both the activated coagulation factor IX and factor X, was developed to decrease the bleeding rate in hemophilia A patients. It bridges the activated factor IX (factor IXa) and factor X, hence facilitating the intact cascade reaction. A phase I clinical study was conducted in healthy adults and in patients by single subcutaneous infusion of emicizumab [97]. The median annual bleeding rates were significantly reduced. Additionally, it shows a high subcutaneous bioavailability with a prolonged plasma half-life [98].

Wnt signaling is critical to promote osteoblast genesis and bone formation that occur during growth, bone homoeostasis or fracture repair. Sclerostin and Dickkopf-1 (DKK-1) are secreted factors that block Wnt signaling. A bispecific IgG against DKK-1 and sclerostin is constructed to treat and prevent bone disease, offering a promising therapeutic approach to the low bone mass disorders like osteoporosis. This bispecific IgG finally showed excellent bone repairing activity compared with parent mAb combinations [99].

*Pseudomonas aeruginosa* is a common opportunistic pathogen with high drug resistance and poor clinical prognosis. Two antigenic proteins, PcrV and Psl, have been shown to play an important role in acute and chronic *Pseudomonas aeruginosa* infection. The patients who were infected generally lack pre-existing immunity and cannot initiate an effective humoral response to these two antigens [100]. Therapeutic strategies against PcrV and Psl are a promising method for the prevention of bloodstream infections of *Pseudomonas aeruginosa*. A new bispecific antibody targeting PcrV and Psl (MEDI3902) has been reported [101]. Compared with the parent monoclonal antibody, MEDI3902 was proven to have synergistic protective activity in the mouse model, which was considered to stem from enhanced targeting of the anti-PcrV arm via binding to the abundant surface Psl. Due to the outstanding effect of the bispecific antibody in preclinical experiments, it is the first bispecific antibody to enter clinical testing against a bacterial pathogen. MEDI3902 is currently under evaluation for safety and pharmacokinetics in healthy adults [101].

The blood-brain barrier (BBB) is the main obstruction hampering the treatment of monoclonal antibodies that have therapeutic potential for treating diseases of the central nervous system. A bispecific antibody was designed to bind transferrin receptor (TfR) combined with a binding site directed against a target BACE1 in the brain. BACE1 is an aspartyl protease responsible for the accumulation of amyloid-β (Aβ) peptides, which are associated with Alzheimer’s disease. Inhibition of BACE1 also provides an ideal signal for the activity of antibodies that cross the BBB. These bispecific antibodies with low affinity of binding to the TfRs can successfully cross the BBB and reduce brain amyloid-b (Ab) in mice, as well as nonhuman primates [102].

Broadly neutralizing antibodies (bNAbs) against the HIV-1 envelope glycoprotein (Env) have exhibited extraordinary potency. Nonhuman primate studies have shown that HIV envelope antibodies can prevent viral infection and control viremia; while because of the high mutant rate, co-administration of different bNAbs is needed to target distinct epitopes. Recently, there have been some bispecific antibodies with exquisite potency against HIV-1, which show great ability for HIV prevention and treatment [103,104].

## 4. Conclusions

As two bispecific antibodies have gained marketing approval, with increasing ones entering clinical trials, bispecific antibodies have already shown great potential not only for tumors, but also for other diseases. It can be predicted that there will be more bispecific antibodies entering clinical development and finally getting approval. This will attract attention from industry and the investment community. While the protein expression, stability issues, the application problems and the use of standard approaches for manufacturing are also as important as the structure design and need to be solved, standard antibody platforms need to be improved and applied in order to put forward the application of bispecific antibodies [105].

Bispecific antibodies are also a platform for researchers to work on their own ideas. The current development of bispecific antibodies is focusing mainly on recruiting immune cells, blocking signaling pathways and delivering toxicities. We can continue to introduce our innovative ideas to this field, as well as introduce some already existing methods, like ADCs [39]. It will be valid and promising to combine antibodies against tumor angiogenesis with antibodies against tumor cells, wherein simultaneously blocking the angiogenesis and tumor cell growth seems to inhibit the tumor progression completely.

## Figures and Tables

**Table 1:** Clinical trials about bispecific antibodies.

Molecule	Targets	Format	Status of Clinical Trail	Disease	Citation
Blinatumomab	CD19 + CD3	BiTE	approved	Acute lymphoblastic leukemia	[8,44,45,46]
MT-110	EpCAM + CD3	BiTE	Phase I (completed)	Lung, gastric, colorectal, breast, hormone-refractory prostate cancer, and ovarian cancer	[47] (ClinicalTrials.gov: NCT00635596)
MT-111 (MEDI-565/AMG 211)	CEA + CD3	BiTE	Phase I (completed)	Gastrointestinal adenocarcinomas	[48,49] (ClinicalTrials.gov: NCT01284231)
AMG 112 (BAY2010112)	PSMA + CD3	BiTE	Phase I (recruiting)	Prostate cancer	[50] (ClinicalTrials.gov: NCT01723475)
MGD006	CD123 + CD3	DARTs	Phase I (recruiting)	Acute myeloid leukemia	[51] (ClinicalTrials.gov identifier: NCT02152956)
AFM11	CD19 + CD3	TandAbs	Phase I (recruiting)	Relapsed and/or refractory CD19-positive B-cell NHL	[14] (ClinicalTrials.gov: NCT02106091)
AFM13	CD30 + CD16A	TandAbs	Phase II (recruiting)	Hodgkin lymphoma	[52] (ClinicalTrials.gov: NCT02321592, NCT01221571, NCT02665650)
CEA TCB	CEA + CD3	Knobs into Holes, CrossMAb	Phase I (recruiting)	Locally advanced and/or metastatic solid tumors	[17,38] (ClinicalTrials.gov: NCT02324257)
catumaxomab	EpCAM + CD3	quadroma	Approved	Malignant ascites (MA)	[53,54]
ertumaxomab	HER2 + CD3	quadroma	Phase II	Breast cancer	[55] (clinicaltrials.gov: NCT00351858)
FBTA05	CD20 + CD3	quadroma	Phase I/II	NHL	[56] (clinicaltrials.gov: NCT01138579)
MM-111	HER2 + HER3	(scFv)_2_-human serum albumin	Phase I	HER2-positive tumors	[20] (ClinicalTrials.gov: NCT00911898,NCT01304784)
DT2219 and DT2219ARL	CD22 + CD19	(scFv)_2_	Phase I	B-lineage leukemia or lymphoma	[57,58] (ClinicalTrials.gov: NCT02370160, NCT00889408)
